# Long-lasting Relaxation of Corrugator Supercilii Muscle Contraction Induced by Near Infrared Irradiation

**Published:** 2011-02-16

**Authors:** Yohei Tanaka, Kiyoshi Matsuo, Shunsuke Yuzuriha

**Affiliations:** ^a^Clinica Tanaka Anti-Aging Center, Matsumoto, Nagano 390-0874, Japan; ^b^Department of Plastic and Reconstructive Surgery, Shinshu University School of Medicine, Matsumoto, Nagano 390-8621, Japan

## Abstract

**Objective:** We previously reported that near infrared (NIR) irradiation weakens frontalis muscle contraction resulting in brow ptosis. In addition, NIR irradiation non-thermally long-lasting thinning of the panniculus carnosus muscle in rats. We sought to determine whether NIR irradiation affects contraction of the corrugator supercilii muscle (CSM). **Methods:** Three to 5 rounds of NIR irradiation were performed weekly to relax the CSM in 40 patients who had sustained myalgia of the CSM. Each round of irradiation consisted of 10 to 40 doses of NIR irradiation at 28 to 40 J/cm^2^. We measured changes in the location of the most medial point of the eyebrow rather than evaluation of the glabellar frown line, especially in the 10 unilaterally irradiated patients. We confirmed relief of the CSM myalgia, using a visual analog scale (10 cm in length) in 40 patients. **Results:** The location of the most medial point of the eyebrow was significantly and durably displaced in the lateral direction when compared with that of the non-irradiated eyebrow, especially in the unilaterally irradiated patients. The visual analog scale score improved from 10 cm to approximately 3 cm in the 40 patients treated. **Conclusions:** NIR irradiation appeared to induce long-lasting relaxation of the CSM, resulting in the lateral displacement of the most medial point of the eyebrow and relief of its myalgia. Although NIR irradiation flattens skin wrinkles by dermal thickening, it may also non-thermally relax the underlying superficial muscles to reduce wrinkles and myalgia.

We previously reported that near infrared (NIR) irradiation used at specific wavelengths accompanied with contact cooling to flatten wrinkles causes a thickening of the dermis. This treatment increases the amount of water retained in the dermis by inducing the expression of collagen, elastin, and water-binding proteins, which protects the subcutaneous tissues from damages due to NIR irradiation.^1^,^2^ Our findings also suggest that specialized NIR irradiation weakens frontalis muscle contraction through the skin resulting in brow ptosis, induces durable thinning of the panniculus carnosus muscle in rats,^3^ and mediates a non-thermal cytocidal effect on cancer cells in mice,^4^ most likely due to apoptosis. Therefore, we sought to determine whether NIR irradiation can affect contraction of the corrugator supercilii muscle (CSM) through the skin.

## MATERIALS AND METHODS

### NIR device and irradiation

NIR irradiation was generated with a broadband infrared source (Titan; Cutera, Brisbane, Calif). The device emits NIR spectrum with a range of 1100 to 1800 nm and filters wavelengths between 1400 and 1500 nm. This device delivers NIR wavelengths that are not strongly absorbed by water or hemoglobin and allows for the safe delivery of NIR irradiation into the deep tissues. The system delivers energy with a fluence range of 5 to 56 J/cm^2^ using continuous-energy single-irradiation pulses of 4 to 10 seconds. A sapphire contact cooling tip was set to a fixed temperature of 20°C to provide contact cooling. Pre-, parallel-, and postcooling of the superficial layers was accomplished using a temperature-controlled sapphire window, which further prevented excessive superficial heating.

**Figure 1:**
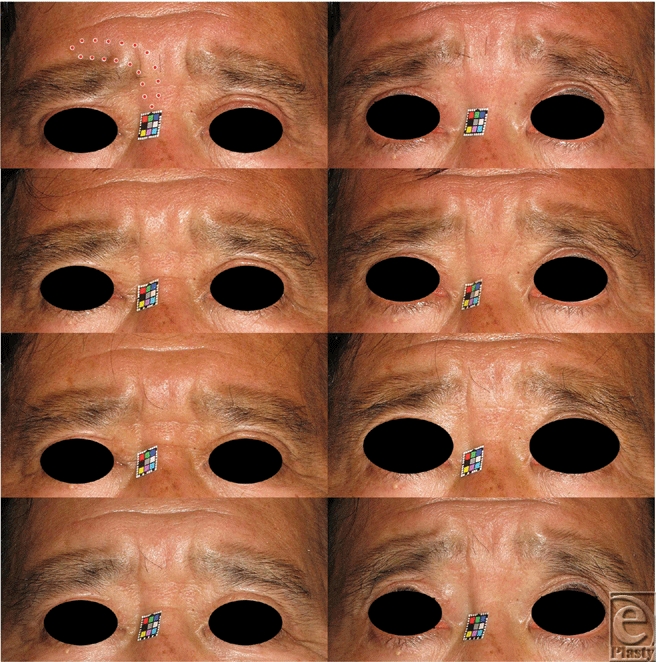
A 57-year-old man who sustained glabellar frown lines and myalgia of the right corrugator supercilii muscle. (*Abov*e) Primary and upward gazing before irradiation. The area surrounded by a dotted line indicates the unilaterally irradiated area. Five rounds of NIR irradiation were performed every week. One round consisted of 20 shots of NIR irradiation at 40 J/cm^2^. (*Above center*) Thirty days after irradiation. (*Below center*) Ninety days after irradiation. (*Belo*w) Three hundred sixty days after irradiation.

**Figure 2:**
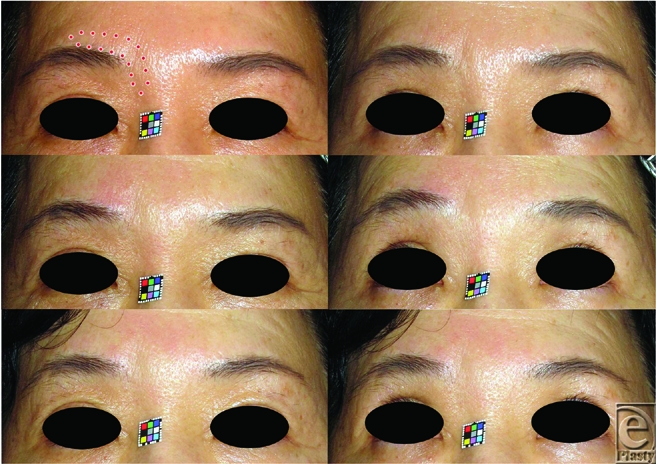
A 60-year-old woman who sustained a right glabellar frown line and myalgia of the right corrugator supercilii muscle (CSM) and was irradiated unilaterally. (*Abov*e) Primary and upward gazing before irradiation. The area surrounded by a dotted line indicates the unilaterally irradiated area. Two rounds of NIR irradiation were performed every week. One round consisted of 35 shots of NIR irradiation at 40 /cm^2^. (*Center*) Immediately after irradiation. The most medial point of the right eyebrow appeared to be dislocated laterally. (*Belo*w) Ninety days after irradiation.

NIR irradiation was performed to flatten glabellar frown lines in the regions of excessive CSM contraction, avoiding the eyebrow in order to reduce possibility of hair follicles damage (Fig 1 *above left* and Fig 2 *above left*). Three to 5 rounds of NIR irradiation were performed each week. Each round of irradiation consisted of 10 to 40 doses of NIR irradiation at 28 to 40 J/cm^2^. Before each dose, additional contact cooling was performed using ice packs.

Fluence ranges for the study were determined by clinical experience with the specialized NIR device and prior published efficacy ranges.^1^,^2^ Typical irradiation parameters ranged from 22 to 40 J/cm^2^. We have previously reported that NIR irradiation at 20 J/cm^2^ is sufficient to induce histological changes in the epidermis; however, higher energies have a greater response and are preferable for dermal effects such as skin tightening.^1^,^2^ Therefore, we irradiated patients at the highest energy (28 to 40 J/cm^2^ that could be used without inducing topical anesthesia.

### Patients and evaluations

We evaluated 40 Japanese patients (34 women and 6 men) ranging from 25 to 67 years of age (46.82 ± 14.27 years), who visited the Clinica Tanaka Anti-Aging Center and had sustained glabellar frown lines and myalgia of the CSM. We excluded patients who had previously undergone botulinum toxin treatment. Ten of the 40 patients were irradiated at the unilateral CSM and the remaining were irradiated at the bilateral CSMs. Patients received a follow-up examination for a period ranging from 90 to 360 days (214.3 ± 50.5 days) after the final irradiation.

**Figure 3:**
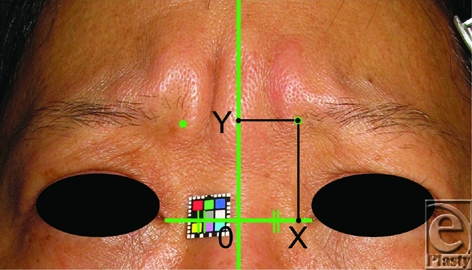
A method for measuring the location of the most medial point of the eyebrow. A horizontal line between both medial canthi was defined as the X axis and a vertical line on the middle point of the horizontal line was defined as the Y axis. We measured X and Y components of the most medial point of each eyebrow on the irradiated and non-irradiated side before and after irradiation.

To evaluate changes in contraction of the CSM, we measured the location of the most medial point of the eyebrow during primary and upward gazing before and after irradiation of the 10 unilaterally irradiated patients. We chose a horizontal line between the medial canthi as the X axis and a vertical line that bisected the horizontal line as the Y axis (Fig 3). We measured X and Y co-ordinates of the most medial point of the eyebrow and calculated the relative ratio between the pre- and post-irradiation co-ordinates on X and Y axes as X post/X pre and Y post/Y pre. Each of these co-ordinates was calculated by dividing the post-irradiation value by the pre-irradiation value, respectively.

In this study, *myalgia* was defined as the existence of muscle aches and tenderness. To evaluate relief of myalgia in the CSM, we compared visual analog scales (10 cm in length) before and after NIR irradiation in all 40 patients.

All measurements were made on photographs using a 10-mm square scale (Casmatch; Dai Nippon Printing Co, Ltd, Tokyo, Japan), which was attached to the skin near the medial canthus of the dominant eye. All patients gave informed consent to participate in the study, which was approved by our institutional review board for human subjects.

### Statistical analyses

The X post/X pre and Y post/Y pre ratios between the irradiated and non-irradiated sides in the 10 unilaterally irradiated patients were analyzed using the Wilcoxon signed rank test. *P* < .05 was used to indicate statistical significance. Data are represented as mean ± standard deviation (SD).

## RESULT

The means and SDs of the X and Y co-ordinates of the most medial point of the eyebrow of 10 patients in primary or upward gazing before and after irradiation were tabulated (Table 1). As a control, we also measured similar co-ordinates in the same patients on the side of the face that was not irradiated (Table 1)

**Figure 4:**
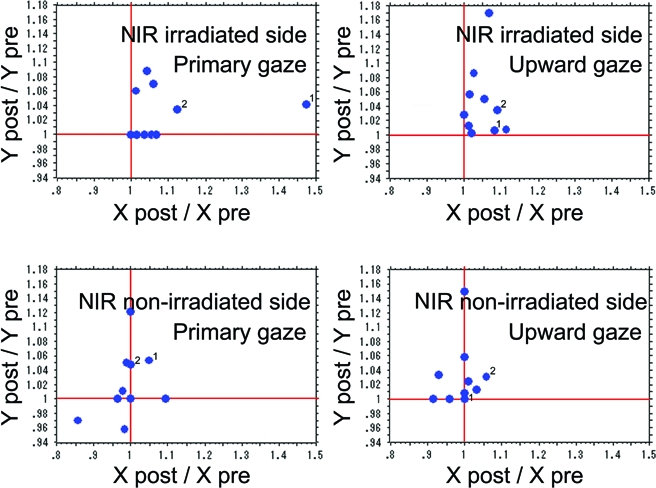
Relative displacements of the most medial point of the eyebrow after NIR irradiation in the 10 unilaterally irradiated patients. The X axis indicates the relative ratio between the pre- and post-irradiation X components X post/X pre. The Y axis indicates the relative ratio between the pre- and post-irradiation Y components (Y post/Y pre). (*Above*) The relative displacements of the most medial point of the eyebrow on the NIR irradiated side during primary gazing (*left*) and upward gazing (*right*). (*Below*) Those on the NIR non-irradiated side during primary gazing (*left*) and upward gazing (*right*). The “1” and “2” indicates the cases shown in Figures 1 and 2, respectively.

The mean X post/X pre ratio on the irradiated side during primary gazing (Fig 4 *above left*), the non-irradiated side during primary gazing (Fig 4 *below left*), the irradiated side during upward gazing (Fig 4 *above right*), and the non-irradiated side during upward gazing (Fig 4 *below right*) were calculated. The mean Y post/Y pre ratio on the irradiated side during primary gazing (Fig 4 *above left*), the non-irradiated side during primary gazing (Fig 4 *below left*), the irradiated side during upward gazing (Fig 4 *above right*), and the non-irradiated side during upward gazing (Fig 4 *below right*) were calculated. The ratios and SDs are shown in Table 2.

Significant differences were observed in the X post/X pre ratios between the irradiated side and the non-irradiated side during both primary (Fig 4 *left*; *P* = .0410) and upward gazing (Fig 4 *right*; *P* = .0011). However, significant differences were not observed in the Y post/Y pre ratios between the irradiated side and the non-irradiated side during both primary gazing (Fig 4 *left*; *P* = .3941) and upward gazing (Fig 4 *right*; *P* = .0966).

The visual analog scale score improved from 10 to 3.0 ± 1.9 cm in 40 patients.

None of the 40 patients who were irradiated have suffered any apparent complications due to the NIR irradiation.

## DISCUSSION

NIR irradiation induced a long-lasting relaxation of the CSM and resulted in lateral displacement of the most medial point of the eyebrow and subsequent relief of myalgia.

The CSM originates vertically from a bony plateau on the supraciliary arch and travels laterally, with a majority of the muscle passing through the fibers of the orbicularis oculi and the frontalis.^5^ The muscle inserts within the dermis above the central portion of the eyebrow. Contraction of the CSM draws the eyebrow medially and caudally, producing a vertical wrinkle at the glabellar area.^6^ We avoided NIR irradiation on the medial eyebrow to reduce possibility of hair follicles damage. The vertical portion of the CSM (in the vicinity of its origin) is located deep on the bone and weakening of the CSM would not occur in the cephalic direction. Therefore, the significant increase in cephalic displacement of the most medial point of the eyebrow upon irradiation may not occur during primary or upward gazing.

In this study, we assumed that myalgia of the CSM is caused by increased contraction. This supraciliary myaglia can be interpreted as a trigeminal neuralgia of the supraorbital nerve or a migraine wherein the myalgia is pulsating. Injection of botulinum toxin^7^,^8^ or lidocaine^9^ to the supraciliary zone may not relieve the neuralgia, but rather relaxes contraction of the CSM. It is unknown why contraction of the CSM is increased resulting in glabellar frown line and myalgia. The CSM is often regarded as the principal muscle involved in the expression of suffering. In addition, the CSM contracts to prevent glare from the sun by pulling the eyebrows toward the bridge of the nose thereby making a roof above the inner corners of the eye. As a person ages, the CSM may become hyperactive and deepen the glabellar frown lines in a similar manner to how the hyperactive levator labii superioris alaque nasi muscle deepens the medial nasolabial fold.^10^

NIR spectrum ranges between visible light and microwave emissions. The NIR is an electromagnetic wave that simultaneously exhibits both wave and particle properties and is known to be absorbed by water and hemoglobin. As a consequence, NIR irradiation is used to noninvasively measure the amount of oxyhemoglobin and deoxyhemoglobin as a pulse oximeter. Hemoglobin is a conjugated protein containing four heme groups and four globins, whereas myoglobin is a conjugated protein resembling a single subunit of hemoglobin. Because both hemoglobin and myoglobin absorb the NIR,^11^ non-thermal damage to myoglobin might explain the effect of NIR on the superficial skeletal muscles, including the CSM and frontalis muscle in humans and the panniculus carnosus muscle in rats.^3^,^4^

As NIR radiation from the sun is selectively filtered by atmospheric water,^12^,^13^ it resembles the NIR device used in this study. Therefore, most NIR radiation that reaches the earth's surface readily penetrates the skin^12^ and may possibly induce non-thermal effects on the subcutaneous tissues as an electromagnetic wave. The total incident solar energy at sea level in North America is 0.0747 W/cm^2^.^13^ One average dose of NIR irradiation onto the CSM consisted of approximately 10 to 40 doses at 28 to 40 J/cm^2^, which was equivalent to the energy received by a few hours of sunbathing in North America.

## CONCLUSIONS

NIR irradiation appeared to not only thicken the dermis but also non-thermally relax the CSM. This simple technique may offer an alternative method to relax superficial facial muscle contraction, which causes facial wrinkles and myalgia. In contrast, solar NIR radiation may also cause unexpected superficial muscle relaxation in areas of the body that are exposed to the sun. Therefore, exposed skin should be protected with sunscreen that blocks NIR radiation to prevent overlying skin ptosis. Additional non-thermal studies are required to decipher how superficial muscle relaxation is induced in humans.

## Acknowledgments

We thank the members of Cutera Inc. to for helpful comments.

## Figures and Tables

**Table 1:** The means and SDs of the X and Y co-ordinates of the most medial point of the eyebrow of 10 patients in primary or upward gaze before (pre) and after (post) irradiation on the irradiated (IR) and non-irradiated (NI) sides

	X-pre	X-post	Y-pre	Y-post
Primary—IR	14.5 ± 3.2	15.4 ± 2.9	22.0 ± 4.9	22.6 ± 4.5
Primary—NI	16.5 ± 4.2	16.2 ± 4.0	23.8 ± 5.1	24.2 ± 4.9
Upward—IR	15.1 ± 3.1	15.8 ± 3.1	24.7 ± 6.4	25.8 ± 6.8
Upward—NI	16.8 ± 4.1	16.6 ± 4.0	26.1 ± 6.1	26.9 ± 6.4

**Table 2:** The relative ratios and SDs between the pre- and post-irradiation co-ordinates on X and Y axes of the most medial point of the eyebrow of 10 patients in primary or upward gaze on the irradiated (IR) and non-irradiated (NI) side

	X-post/X-pre	Y-post/Y-pre
Primary—IR	1.089 ± 0.140	1.030 ± 0.035
Primary—NI	0.991 ± 0.061	1.021 ± 0.048
Upward—IR	1.048 ± 0.039	1.045 ± 0.051
Upward—NI	0.990 ± 0.044	1.032 ± 0.045
